# Chromhome: A rich internet application for accessing comparative chromosome homology maps

**DOI:** 10.1186/1471-2105-9-168

**Published:** 2008-03-26

**Authors:** Sridevi Nagarajan, Willem Rens, James Stalker, Tony Cox, Malcolm A Ferguson-Smith

**Affiliations:** 1 Cambridge Resource Centre for Comparative Genomics, Department of Veterinary Medicine, University of Cambridge, Madingley Road Cambridge CB3 0ES, UK; 2 The Wellcome Trust Sanger Institute, Wellcome Trust Genome Campus, Hinxton, Cambridgeshire CB10 1SA, UK

## Abstract

**Background:**

Comparative genomics has become a significant research area in recent years, following the availability of a number of sequenced genomes. The comparison of genomes is of great importance in the analysis of functionally important genome regions. It can also be used to understand the phylogenetic relationships of species and the mechanisms leading to rearrangement of karyotypes during evolution. Many species have been studied at the cytogenetic level by cross species chromosome painting. With the large amount of such information, it has become vital to computerize the data and make them accessible worldwide. Chromhome  is a comprehensive web application that is designed to provide cytogenetic comparisons among species and to fulfil this need.

**Results:**

The Chromhome application architecture is multi-tiered with an interactive client layer, business logic and database layers. Enterprise java platform with open source framework OpenLaszlo is used to implement the Rich Internet Chromhome Application. Cross species comparative mapping raw data are collected and the processed information is stored into MySQL Chromhome database. Chromhome Release 1.0 contains 109 homology maps from 51 species. The data cover species from 14 orders and 30 families. The homology map displays all the chromosomes of the compared species as one image, making comparisons among species easier. Inferred data also provides maps of homologous regions that could serve as a guideline for researchers involved in phylogenetic or evolution based studies.

**Conclusion:**

Chromhome provides a useful resource for comparative genomics, holding graphical homology maps of a wide range of species. It brings together cytogenetic data of many genomes under one roof. Inferred painting can often determine the chromosomal homologous regions between two species, if each has been compared with a common third species. Inferred painting greatly reduces the need to map entire genomes and helps focus only on relevant regions of the chromosomes of the species under study. Future releases of Chromhome will accommodate more species and their respective gene and BAC maps, in addition to chromosome painting data. Chromhome application provides a single-page interface (SPI) with desktop style layout, delivering a better and richer user experience.

## Background

Comparisons between species genomes are based on maps, which vary in resolution from gene rich maps of human or mouse to gene poor maps of other mammalian species. Numerous online resources are available for comparing sequenced genomes. Ensembl [1], NCBI [2] and Human Genome Browser [3] are large databases holding genomic as well as comparative sequence information for mammalian species. Mouse Genome Database, Rat Genome Database, Flybase and Wormbase are species specific databases. Bioinformatics tools like PipMaker [4], VISTA [5], BLAST and CLUSTAL-W offer local and global alignments of two or more genomic sequences. These resources make comparisons and predictions of phylogenetic relationships easy and accurate for sequenced species.

From the evolutionary point of view, representative species of all families and orders are important, as they can provide insights into phylogenetic relationships. Sequencing these genomes would be a considerable task for biologists. It is therefore essential to have basic first hand information about species genomes through simpler and faster methodologies. Comparative chromosome painting can be used here, as a guide to the construction of simple maps in unmapped species or as a useful tool for studying karyotype evolution. When chromosome specific paint probes from one species (probe species) are hybridised to the chromosome preparations from another species (target species), homologous regions conserved between the two species are detected [6] The resolution is estimated to be approximately between 2 Mb to 3 Mb. Reciprocal cross-species painting helps to determine the genetic content and position of the homologous regions of one species on the other. This information is valuable in identifying ancient syntenies shared by widely divergent species and in indicating the likely structure of ancestral chromosomes. When large blocks of homology are associated with one another in all species of a particular taxonomy they are regarded as being shared ancestral characters. Other syntenies can be considered as common derived characters because they have arisen in a known common ancestor and are thus of more recent origin.

Human chromosome paint probes have provided the evidence for the conservation of such syntenies across mammalian orders and for the construction of presumptive ancestral karyotypes [6]. Chromosome homology maps can also be used to aid comparative gene mapping. The location of EST or genes of interest in an unmapped species is most likely to be found within the region that shares homology with the human segment that contains the orthologous sequence.

With the great volume of cross-species painting data, there is a need for a flexible, scalable, readily available, and user-friendly software application for storing and comparing the chromosome information of various species. **CHROMHOME **(**Chrom**osome **Ho**mology **M**apping **and E**lectronic-painting) is a comprehensive web application that is designed to provide cytogenetic comparisons among species using chromosome painting.

## Implementation

### Data Collection

**Figure 1:**
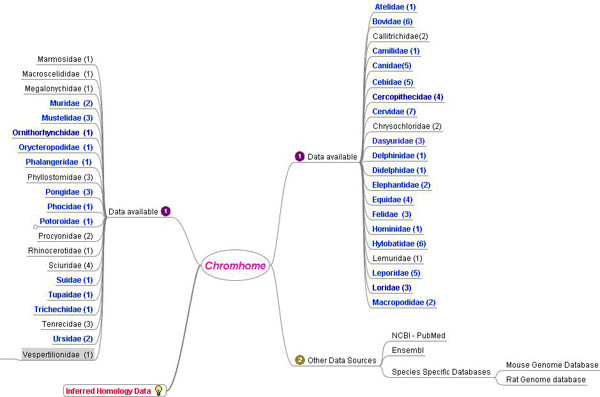
**Data resources for Chromhome**. Molecular Cytogenetics group at Cambridge Resource centre, CRC (1) has published data for 100 species from 42 families. The blue coloured families represent at least one species from the family on Chromhome. External data resources (2) are through NCBI Pubmed searches for cross species painting data, Ensembl for karyotype information for sequenced species and specific species genome databases such as Mouse Genome Database or Rat Genome Database.

Various research groups from around the world have generated cross species painting data from representative species of all mammalian orders [7-15]. Cross species chromosome painting data is available for over 100 species from different families of mammals, birds and reptiles (Figure 1), and can be obtained through Pubmed searches. For species that do not have sequence data available in online databases, the only sources for initial cytogenetic data acquisition are publications. Although a wide range of literature exists, for each species the entire cytogenetic data may not appear in the same publication. The painting data, the reverse painting and the idiograms may occur in different papers. Furthermore, data published by different authors for the same species may differ in their results, which may be due to use of different chromosome nomenclatures. The data from various sources need to be analyzed, reconciled, grouped, processed and made readily accessible.

The data comes in different levels of detail ranging from karyotype to homology maps. G-banding (a technique of staining partially denatured chromosomes with Giemsa stains) forms a pattern of dark and light bands, which enables unequivocal recognition of specific chromosomes. An idiogram is a graphical representation of a G-banded karyotype. Species like human or mouse have G-banded idiograms and related sequence information that can be obtained from Ensembl. But most other species have only G-banded karyotypes. The Atlas of Mammalian Chromosomes [16] holds karyotype data for 815 species of which 43 have idiograms that are used in Chromhome.

### Data Storage and Processing

Chromhome uses a Relational Database Management System (RDBMS), as it offers structured storage and maintains data integrity. The database of choice for Chromhome is MySQL [17]. Retrieval of information from the database is through a series of SQL scripts and the result-set can be exported easily in text formats. This database forms the central repository for overall Chromhome data organization giving the added benefits of concurrency, scalability and transaction supports. The techniques to integrate the database to feed web applications use JDBC (Java Data Base Connectivity) protocols and related java APIs (Application Programming Interface) wrapped in OR/M (Object Relational Mapping) implementations.

To convert the pictorial data to numerical genome based data for Chromhome, the total genome size in basepairs is used to calculate the approximate size of each chromosome in a species. In the Animal Genome Size Database, Release 2.0 [18] the genome size, or the C-value, for 4379 species is published. The C-value is expressed in picograms and can be converted to the number of nucleotides in the genome by an established formula [19]. For species with no genome size information, the genome size of a closely related species or species from the same family is considered. Based on the measurements of the lengths of chromosomes and size of chromosome homologous regions, an idiogram and homology maps are created for each species. The data are presented as G-banded karyotypes or idiograms for the species. In the homology maps, probe chromosomes are depicted as blocks on chromosomes of the target species to show homologies between them.

### Chromhome Architecture

**Figure 2:**
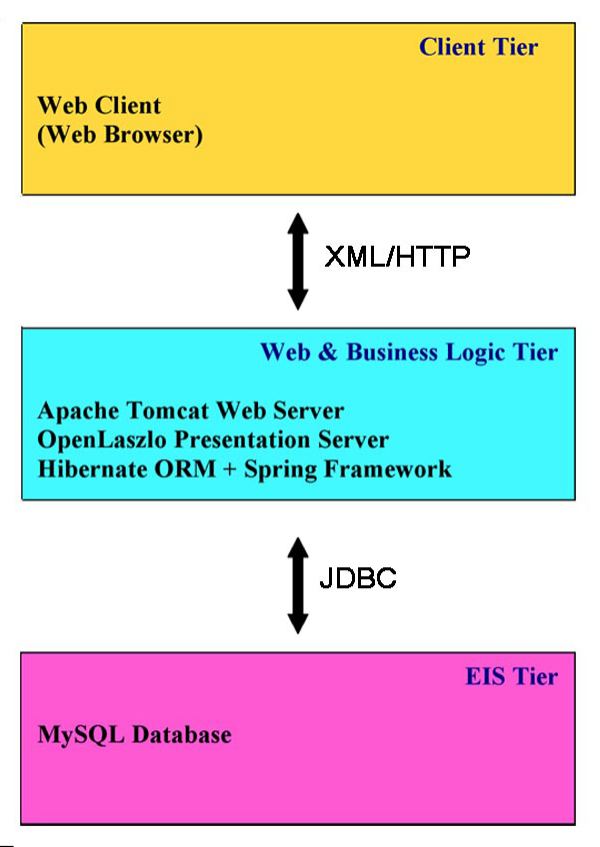
**Chromhome n-tier architecture**. The Web Client layer is the web browser layer through which the user selects the target, probe and method species combination and sends a http request. The request goes through the middle tier that connects to the database layer through JDBC (Java Database Connectivity) and retrieves the information from the database. The data is processed in the middle tier through a series of programs and is sent back to the web browser, where the client can view the homology maps.

Software system architecting consists of deciding what tiers are needed for the application and what services are required at each tier. The Chromhome application is designed and architected in accordance with separation of concerns and design-by-contract (DBC) principles of software best practices [20]. Accordingly, the application architecture forms the n-tier enterprise model (Figure 2) with an interactive client layer, presentation layer, business logic layer and the data layer. The Java Enterprise Edition (JEE) is the underlying technology stack. A number of industry-standard open source solutions are employed to build Chromhome.

OpenLaszlo [21] Rich Internet Application (RIA) platform is used for the development and deployment of the web pages. The presentation pages of Chromhome are written with OpenLaszlo's markup language LZX (Laszlo XML). These are saved on the server side and invoked by clients in the runtime. The server component is deployed as a J2EE web application (a servlet) that intercepts the script requests from the client, interprets them, and serves them as Flash runtime binaries. Apache Jakarta Tomcat [22] is used as the web server for Chromhome application. Tomcat along with OpenLaszlo provide the presentation tier. Hibernate [23] Object-Relational Mapping (O/RM) tool is used to develop persistent classes for Chromhome following Java idioms like association and composition. All the Chromhome domain classes are implemented as POJOs (Plain Old Java Object). The instance variables in the class are mapped with the columns in the table. Combining Spring Framework [24] with Hibernate greatly reduces the amount of plumbing code. Chromhome uses Spring DAO (Data Access Object) support for Hibernate. Spring and Hibernate frameworks support the business logic tier of Chromhome application.

**Figure 3:**
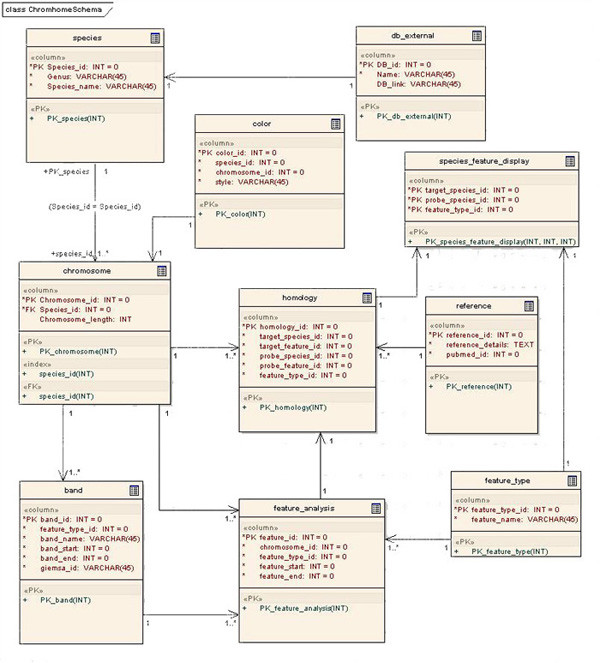
Chromhome Data Model diagram represents the schema with entity-relationship multiplicity.

The whole project is tailored to follow Rational Unified Process (RUP) with use case driven, architectural-centric and iterative ingredients. During the initiation phase of the project, the important use cases were identified and the event flows were documented. Use case realizations were achieved through sequence diagrams. UML (Unified Modelling Language) [25] modelling is done through the Enterprise Architecture [20] design tool. The Database Model describes the data which must be stored and retrieved as part of the overall Chromhome design. This describes the tables and data in detail and enables generation of DDL (Data Definition Language) scripts to create and setup databases. Chromhome Data Model diagram (Figure 3) represents the schema with entity-relationship multiplicity.

**Figure 4:**
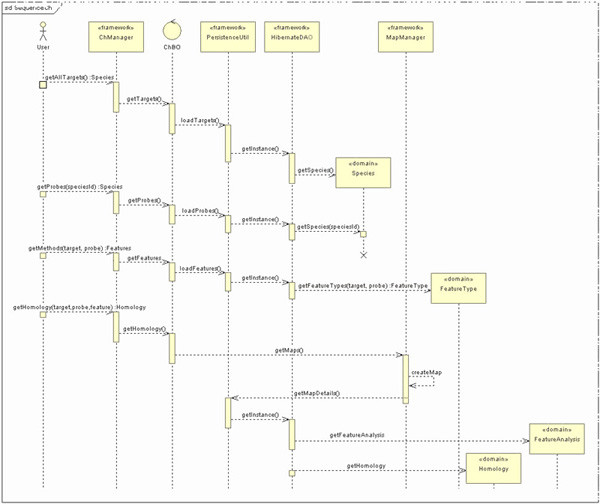
Sequence diagram of Chromhome.

The main sequence diagram of Chromhome is shown in Figure 4. The initial user request is handled by the java class, ChManager. ChManager serves as a façade class for the application. It communicates with the business object class ChBO, to fetch the requested data. Business object class wraps all the application logic and through the data utility class, PersistenceUtil, it organizes the data retrieval. Species, FeatureType, Homology and FeatureAnalysis are some of the domain classes that are mapped to database tables. MapManager component handles the idiogram and homology map generation. MapManager gets the data in XML format and makes use of Ensembl draw code. Eclipse platform [26] with MyEclipse [27] Java EE IDE (Integrated Development Environment) is used to code, debug and unit test the application

## Results and Discussion

**Figure 5:**
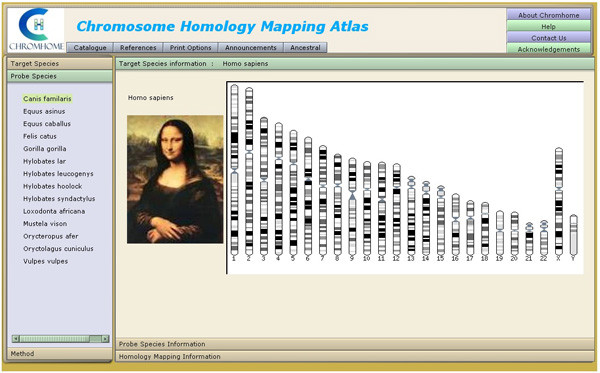
Screen shot of Chromhome from [29].

**Figure 6:**
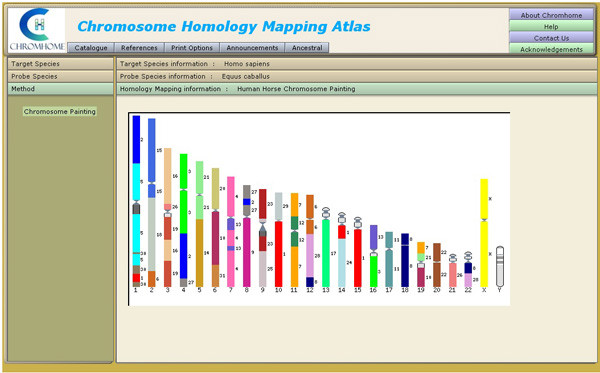
Screen shot for Homology Map displaying the horse homologies on human chromosomes.

Chromhome Release 1.0 houses 109 chromosome comparative maps and G-banded idiograms of 51 species. The initial selection view is shown in Figure 5. The screen represents two views. The view on the left provides easy to use UI (User Interface) elements to facilitate effective user interaction with the application. The user can select target and probe species and the method to get the chromosome maps. The view on the right presents the resulting homology maps and species information (Figure 6).

The selection view on the left presents two sets of species on two separate tabbed views. The target is the species on whose chromosomes the probe species chromosomes are hybridized to obtain the chromosome homology maps. Selecting the target species through a mouse click brings up another tab view presenting a set of probe species that has homology data with the selected target species. Further selection of probe species presents another tab view of methods available for the given target-probe combination. When the user selects a method, the homology map is displayed on the right, for that unique target-probe-method combination. In the homology map, the probe species chromosomes are marked along the sides of the target species chromosomes. Each probe chromosome is assigned a specific colour for ease of identification and possible pattern recognition within the homology map. There are menu options to print homology maps and idiograms. The references for target, probe and homology data are available in the reference menu. PubMed ids to the references are provided and displayed on the reference window. The catalogue menu shows the available homology maps in Chromhome, in a matrix format.

Inferred painting may help researchers in finding significant information about species where no direct cross-species chromosome painting exists. If species A and species B are mapped on species N, then it is possible to deduce some of the chromosomal arrangements of A on B or B on A with respect to the arrangements of N chromosomes. Many of the species in Chromhome have been mapped on human chromosomes using chromosome painting. It is therefore possible to infer homologies between two species each of which have been hybridized with human probes. In certain cases, inferred homologies result in ambiguities, which are indicated in Chromhome by multiple numbers next to the chromosomes and any one colour pertaining to the probe chromosome is displayed. For instance, ambiguity arises when a chromosome region is conserved between species A and N, but in species B this region is divided in two or more sub regions. An example of the use of a selective gene map of pig to resolve ambiguities in the pig and cow inferred map is seen in Chromhome. A more lengthy discussion on the cause of these ambiguities is beyond the scope of this report and is given elsewhere [Nagarajan et al, in preparation]. Of the 51 species in Chromhome, 22 species have gene/EST/BAC maps available either in genome databases such as Ensembl [1], NCBI [2], and ENCODE [28] or in scientific publications. These maps may be used with homology maps to improve inferred painting results. Chromhome provides inferred paintings for 10 species that do not have chromosome painting data.

In the current release, only chromosome painting by FISH and inferred painting methods are available. Future releases of Chromhome will accommodate gene, BAC and other mapping methods. Features like comparison to ancestral karyotype for each species will also be added. We are working on data and formatting standards to help users to upload their own data and generate homologies. Once the data are published they can be included in Chromhome.

## Conclusion

Chromhome is a rich internet application presenting comparative chromosome homology data for a diverse range of species. Inferred data adds to the value of the application, where one can compare any two species that have been painted with a common third species. This may help researchers to focus on those chromosomes of interest for further analysis. The availability of a number of homology maps for different species on Chromhome provides input for evolutionary analysis and for the prediction of ancestral chromosomes and points us in the direction of functionally important areas of chromosomes in unmapped species.

## Availability and requirements

**Project name**: Chromosome Homology Mapping and E-painting

**Project home page**: Chromhome application is available at: .

**Operating systems**: platform-neutral

**Programming language**: Java

**Other requirements**: The application requires a Web browser with Flash 7, 8, or 9 installed . This includes a wide range of web browsers on a variety of OS across numerous hardware platforms.

Feedback and support queries can be sent to: chromhome@vet.cam.ac.uk.

**Any restrictions to use by non-academics**: None

## Authors' contributions

SN analysed, architected, designed and implemented the database, web interface and the application logic and also drafted the manuscript. JS organised the Ensembl draw code to fit into the Chromhome architecture. TC participated in database design efforts. MAFS and WR guided and coordinated the execution of the project and revised the manuscript. All authors read and approved the final manuscript.
